# The Effects of Adding Different Stabilizers in Brine on the Physicochemical, Sensory, Microbiological and Textural Properties of White Cheese

**DOI:** 10.3390/foods8040133

**Published:** 2019-04-19

**Authors:** Hasan Cankurt

**Affiliations:** Food Technology Department, Safiye Cikrikcioglu Vocational School, Kayseri University, Kayseri 38000, Turkey; hcankurt@erciyes.edu.tr or hcankurt047@gmail.com

**Keywords:** white cheese, cheese storage, thickening agents

## Abstract

The aim of the present study was to determine the effect of four different thickening agents (guar gum, carrageenan, xanthan gum, and gelatin) on the textural and sensory properties of white cheese. For this purpose, various white cheeses were manufactured with brines (the salt ratio was 8% and 12%) that contained different types and concentrations of gums; white cheese textural and sensory properties were studied during storage (at 4 °C). Also, microbiological properties including lactic acid bacteria (LAB) and mesophilic aerobic bacteria were investigated. The physicochemical, textural, microbiological, and sensory properties of the cheeses were determined on the 1st, 15th, and 30th days of storage. During the storage period of cheese, the top-rated samples in terms of sensory properties were cheeses with gelatin and carrageenan gum. The microbiological data displayed that there was an inverse relationship between the number of bacteria and the amount of gum used, although it was not precisely linear. At the same time, the highest values were generally determined in the control samples, and although not to a very great extent, gelatinous examples were found to contain a lower number of lactic acid bacteria than others. Regarding the textural properties, the hardness value of all samples containing gelatin showed a continuous increasing trend, while the springiness values increased only in the samples with xanthan gum and guar gum. At the end of storage, it was observed that the inherent adhesiveness of the samples decreased by more than half and the use of gelatin resulted in an increase in the gumminess of the cheeses. As a result, it was determined that reducing the salt used in the brine by up to 8% did not cause any defect since stabilizers were preventing water passage into the cheese by holding water.

## 1. Introduction

Cheese, which is rich in protein and fat, is a product obtained as a result of the curd being processed in different ways and allowed to mature under certain conditions for certain period of time after the milk is coagulated with rennet and whey is separated. The salting process, which gives the cheese a characteristic taste and appearance and also affects the maturation period except for some varieties consumed fresh and salt-free, is considered as an essential stage of the production. It also affects the proteolysis in cheese and, hence, its structure and taste [1,2,3,4]. White cheese, one of the most common of soft cheeses in Turkey is produced 360 thousand tons annually and is salted in brine.

White cheese in Turkey is usually kept in brine with a salt content of 12–16%. Salt is used to preserve the cheese and give it hardness as well as flavor [5,6]. When the salt ratio is lowered, the cheese tends to melt and eventually softens and dissolves. This is because some minerals that give hardness to the cheese dissolve and pass into the brine, thus the water in the brine moves into the cheese and increases the water content. Brine is prepared with different proportions of salt and various types and amounts of stabilizers are used as a matrix to preserve white cheeses. The main purpose of the brining process is to reduce the movement of water into the cheese by trapping water with stabilizers. In this way, the ratio of salt passing into cheese could also be reduced. Stabilizers were thought to reduce the salt content used in brine (up to 8% salt) by preventing the passage of water into cheese by holding the water [4]. The cheeses are usually kept in brine, or they are salted by sprinkling salt. Besides, there are methods applied in the form of adding salt to milk. The salting process, which is provided by keeping the cheeses in brine containing specific amounts of salt at a specified temperature for a certain period of time, is the most common salting method in terms of (i) ease of application, (ii) regular salt passage, and (iii) the providing the possibility of adjusting the desired salt ratio to the producer [7]. Today, the most common salting method is brine salting. In this process, both the desired taste level of the cheese and low-cost cheese making are achieved [8]. This is not the only reason why salt is essential for cheese technology. The importance of salt is due to many reasons. One of the reasons is that salt gives characteristic flavor to the cheese. Unsalted cheese lacks in taste. To give the cheese flavor, it is sufficient to have a 0.8% salt ratio in the cheese. The salt facilitates the removal of whey from the fresh cheese and helps to establish the water balance. As a result, the water activity of the product is also reduced. Salt affects the operations of rennet, starter, and non-starter lactic acid bacteria (LAB) and their enzymes. Thus, the growth of unwanted bacteria is limited [9].

Salting is very important for the production of cheese with the desired qualities both in terms of legal regulations and consumer wishes. The low or high salt content of cheese negatively affects the quality [10,11]. The salt passage to cheese is explained by the diffusion and osmosis events between cheese and brine. Factors affecting the amount of salt passing to cheese are the surface area of the cheese, the fat content, the acidity, and the salt ratio, temperature, and acidity of brine. If the acidity in the brine is too high, the cheese will have a bitter taste, and if it is too low, it will have a sticky surface and a soapy taste. To avoid these negative effects, the pH value of brine must be between 5.2 and 5.3. However, the closer the pH value of brine and cheese, the more balanced the salt passage becomes [12]. 

Nevertheless, salt consumption in our country is three times higher than the recommended values. Excessive salt consumption leads to high blood pressure. High blood pressure is the leading risk factor of heart disease, which is the most important cause of death and disease burden in our country. It is also closely associated with gastric cancer, osteoporosis, and kidney diseases. Reducing salt consumption gradually to the recommended levels in our society for the protection of public health and prevention of diseases will be possible by changing production techniques and salting methods with a multidisciplinary approach. This study is aimed at the reduction of salt concentrations in the brines used in the production of white cheeses, which are one of the most consumed cheeses all over the world [13].

## 2. Materials and Methods

Platform tests Soxhlet Henkel (SH) (pH, antibiotic, neutralizing agent, Brix, fat ratio, and density) of the milk bought for cheese making were performed, and after concluding that the milk was suitable, the processing started. After the milk was passed through a coarse filtration, it was pasteurized at 80 °C for 1–2 min in a double-walled boiler. It was then immediately cooled down to 37 °C, which is the fermentation temperature, and a 2% pre-activated ready-to-use starter culture (Mystarter Culture CT 201, Strains; *Lactococcus lactis*, *Lactococcus cremoris*, and *Streptococcus thermophiles*, producer: Maysa Food Anonym Co, Istanbul, Turkey) and 1% calcium chloride (CaCl_2_) were added. According to the rennet test, the suitable amount of microbial rennet (Yayla, 1/16,000, liquid rennet, Producer: Maysa Food Anonym Co, Istanbul, Turkey) was added to the cheese and it was fermented with microbial rennet for 1 h. At the end of this period, the curd was broken with curd-breaking wire and waited for 10 min. Then, the whey was drained. After leaving it for half an hour, the curd was collected, and pressure was applied to it. After for two hours, it was cut into 4 × 5 × 7-sized slices. These slices were weighed as 0.5 kg each, and 0.5 kg of brine containing different stabilizers with different ratios and different salt ratios were added to cheese at 30 °C. The brine, which was fluid initially, formed a loose or stable gel when it cooled down. The cheeses were stored in a glass jar at 4 °C throughout the ripening stage. The samples were analyzed on the 1st, 15th, and 30th days. 

Brine with stabilizers was prepared as follows: First, the amount of gum to be used in brine was optimized and was added in the following proportions.

Brine with guar gum: 0.75–0.50% and 0.25% guar gum was weighed and was dissolved in brine containing 8% and 12% salt separately. This brine was pasteurized at 85 °C for 15 min. 

Brine with xanthan gum: 0.75–0.50% and 0.25% xanthan gum was weighed and was dissolved in brine containing 8% and 12% salt separately. This brine was pasteurized at 85 °C for 15 min.

Brine with carrageenan: 1.5–1.0% and 0.75% carrageenan was weighed and was dissolved in brine containing 8% and 12% salt separately. This brine was pasteurized at 85 °C for 15 min.

Brine with gelatin: 2.0–3.0% and 1.0% gelatin was weighed and was dissolved in brine containing 8% and 12% salt separately. This brine was pasteurized at 85 °C for 15 min. 

Control brine: Brine containing 8% and 12% salt was prepared and this brine was pasteurized at 85 °C for 15 min. 

The difference in the ratio of gum in brine was the cause that some of them had a high consistency. Brine with gelatin also had a high consistency. However, they started taking the form of gel and started to become thick after the first day. 

Cheeses were produced in two replications and analyses were carried out in 3 repetitions. 

### 2.1. Textural Properties

The textural properties of cheese samples were determined using the texture analyzer (TA.XT Plus Stable Micro Systems Ltd., Surrey, England) [14]. Samples to be analyzed were cut into cubes of 2.0 cm size. A 2 cm diameter spherical head was used in the pressing. The compression speed was set to 1 mm/s, the total processing time to 10 s, and the compression was carried out to compress 25% of the original size of the samples. According to the texture profile analysis technique, two consecutive compressions were applied, and a tissue profile of samples was determined by measuring tissue profile parameters. In this method, the hardness, adhesiveness, springiness, cohesiveness, gumminess, chewiness, and resilience of the cheese samples were measured. Texture analyses were carried out in 5 repetitions.

### 2.2. Microbiological Properties

LAB and total mesophilic aerobic bacteria (TMAB) counts were performed by using bacteria-counting methods in cheeses. To test the microbial properties of samples, serial dilutions were prepared with 25 g samples and 225 mL sterile ringer solution, and LAB (Streptococcus and lactococci) count and TMAB count were performed. 

LAB found in cheese were determined as colony-forming unit (CFU) in 1 g of cheese at the end of 2–3 days of incubation at 37 °C using De MAN, ROGOSA and SHARPE (MRS) Agar [15,16]. 

TMAB Count was determined at the end of incubation at 30 °C for 24 h using Plate Count Agar (PCA) [17].

### 2.3. Sensory Properties

Sensory analysis was performed according to the hedonic scale method to determine the sensory quality of all cheese types. So, sensory properties of the cheeses were determined by 15 panelists on the 1st, 15th, and 30th days of storage in terms of appearance, taste, texture, and general acceptance from 1 to 9 (1: very bad, 9: very good) [18].

### 2.4. Physicochemical Properties

Total dry matter analysis in cheese was measured by gravimetric method after drying at 105 °C, salt ratios in cheeses were determined made by the Mohr method, and pH analysis was performed with a pH meter (Hanna-Instrument pH microprocessor, Hanna Company, Vinsoket, RI, USA [6].

### 2.5. Statistical Analysis of Data

Statistical analysis of the data obtained was performed using the Windows-based SAS 8.0 statistical package program (SAS, Redmond, DC, USA). The effects of stabilizers and brine concentrations on some properties of cheeses were determined by one-factor and two-factor analysis of variance. The difference between the groups was determined using Tukey’s method for multiple comparisons at *α* = 0.05 significance level [19].

## 3. Results and Discussion

### 3.1. Physicochemical Properties

According to Turkish Standards (TSE), the maximum dry matter ratio in white cheese should be 40%. Table 1 shows that in some cheese samples containing stabilizer, especially in the brine 12%, the dry matter content was over the standards. However, most of the cheese samples contained dry matter less than 40%. In samples containing 8% salt brine, dry matter values decreased after the first day and then increased again. No specific trend was observed cheese samples in brine containing 12% salt. In 8% salted brine samples, the highest total dry matter content was found in the case of carrageenan at the end of storage. In 12% salted brine samples, the highest values were in gelatinous samples. According to the results, white cheese, which are over of the standards, could be standardized by using stabilizer in brine.

The pH values of all samples were lower on the first day of storage and in the 8% brine as shown in Table 2. This is due to the fact that in the cheese samples in brine with 8% the salt content (Table 3) was lower and the moisture content was higher. Samples with 8% salt brine enriched with carrageenan showed the lower pH values throughout storage. In cheese samples kept in 12% salt brine, the lowest pH value was measured in the control sample at the end of storage. It was observed that at 30 days, the pH value of the cheeses kept in brine containing 12% salt and gelatin increased, whereas the corresponding pH values in the 8% brine decreased compared to the first day. 

The results in Table 3 showed that the salt ratio increased during storage in all samples. The aim of the study is to increase the consistency of the brines used in the brine production and to make the salt pass into the white cheese more difficult. However, it did not happen. In all cheese samples with stabilizers, salt content increased throughout storage. This result did not show that the research failed. When the salt content of brine is below 12%, softening occurs in the cheese. In this study, many of the cheeses kept in brine containing stabilizer and 8% salt remained hard, as shown below. However, control cheese softened and melted over time, as expected. The hardness of the control sample containing 12% salt was lower than the hardness value of the cheese kept in brine with 8% salt and 3% gelatin. In addition, the study could be considered successful towards the salt reduction direction because the salt content of the cheese sample kept in brine with 8% salt and 3% gelatin was 4.03%, while salt content of control cheese kept in brine with 12% and 3% gelatin was 5.40% at the end of storage. In other words, while the texture of cheese was preserved, the salt content final cheese product reduced by 25.4%. According to these results, a negative relationship can be established between gelatin ratio and salt ratio.

### 3.2. Microbial Properties

TMAB and LAB counts of cheese samples were analyzed during the study. Although lactococci are the most critical group of LAB in general use, they are used as a starter culture in many fermented milk products, especially in cheese [20,21]. Şengül [22] found the number of Lactococcus in pasteurized milk cheese as 2.3 × 10^6^ CFU/g and the number of Lactococcus in raw cheese as 4.1 × 10^6^ CFU/g. They indicated the addition of starter culture to cheese produced from pasteurized milk as the reason for the high number of Lactococcus. Kesenkas et al. [23], in a study where they examined the village cheeses produced in three different enterprises in the Izmir region, determined the number of Lactococcus as 6.26–7.74 log CFU/g in the first day of the cheeses. Cankurt [7] reported that the number of lactococci measured during the storage of the produced cheeses increased until the 15th day in general and decreased again after the 15th day, and that the increase during storage was statistically significant (Table 4). 

TMAB increased in all samples during storage. The number of bacteria was quite high. At the end of the storage, the highest values were recorded in the cheeses kept in brine with carrageenan.

The effect of stabilizers against lactic acid bacteria, used as the starter culture, was also investigated. When the data were examined, it was seen that there was an inverse relationship between the increase in the amount of gum and the number of bacteria, although it is not entirely linear. It was also seen that the number of LAB of all cheese samples salted with 8% brine increased throughout the storage, except, for example, with 0.5% guar gum. In the first day, the lowest numbers were recorded in the cheese samples with gelatin in brine and the same cheese had the lowest number of LAB at the end of storage. The counts of TMAB were parallel to the counts of LAB; both were found in very high numbers in all samples. It was observed that the use of stabilizers did not have a significant negative effect on starter culture. In general, the highest values were determined in the control cheese samples. Cheeses kept in brine with gelatin were found to contain a lower number of LAB than others, although not in severe proportions (Table 5).

### 3.3. Textural Properties

In this study, the textural properties of white cheese with different stabilizer and salt content in their brine were performed using texture profile analysis (TPA). In this method, the hardness, adhesiveness, springiness, cohesiveness, gumminess, chewiness, and resilience of the cheese samples were measured. 

Hardness is known as the resistance of the product against deformation [24]. Hardness value in literature is usually expressed with g (kg), while Newton (N) is used in some studies [25]. In general, the hardness values of samples kept in brine with 8% salt were increased from 1 to 15 days first, and then decreased. However, the hardness of all cheese samples kept in brine with gelatin increased continuously. There were significant increases in cheeses with brine containing 12% salt. Hardness values increased consistently in cheeses with brine containing 12% salt. A surprising result was that all of the cheeses being in brine with 8% salt enriched in 0.75% guar gum, 0.75% xanthan gum, and gelatin were harder than the control cheese kept in brine with 12% salt. It is understood from here that, despite being in brine with 8% salt, harder but less salty cheeses can be produced compared to the control cheese maintained in brine with 12% salt (Table 6).

The formation of texture in cheese varies with various processes. Some of them are the composition of milk and processing stage of milk [26]. In their studies, Akalın and Karaman [27] found the hardness values of the samples they stored in packaging with brine and vacuum packaging without brine as 1080 g and 1130 g, respectively, at the end of storage. Kaya [28] found the hardness value of Gaziantep cheese in five different brine concentrations, which were 5%, 10%, 15%, 20%, and 25% and salt solutions were as 3.45, 5.67, 10.75, 34.87, and 38.36 N, respectively. In their study, Kırkın et al. [29] found the hardness values between 2.7 and 9.4 N at the end of 13 weeks, and Sahingil et al. [30] found the hardness values as 3.07 N, 4.72 N, and 3.66 N, respectively. Sener reported that [24] the hardness values of control cheeses increased until the 30th day and then did not change until the end of storage in the study where transglutaminase enzyme was used in cheese production. In a study by Cankurt [7], the hardness values of samples during storage were increased first, then decreased, and increased again. Koyuncu showed that [31] the hardness values of the cheeses in his study had been reduced despite the fluctuating course compared to the beginning of storage. The results of our study are consistent with the results in the literature.

Cohesiveness is expressed as the force of intimate bonds between proteins and fats, forming the three-dimensional structure in cheese [24]. When the cohesiveness values of white cheese samples kept in brine with stabilizer were examined, it was seen that the samples exhibited significant changes within themselves and among each other during storage. The cohesiveness values of our samples also followed a fluctuating course like hardness values. Cohesiveness values on the first day were very close to each other in all samples. Although there was no severe decline from the first 15 days at the end of storage, the cohesiveness values of the samples decreased by more than half. While the lowest cohesiveness values were recorded in the control cheeses with brine containing 8% salt, cohesiveness values were among the highest values in cheeses with brine containing 12% salt. The changes in the texture of the cheese during ripening can be explained by the constant breakdown and re-establishment of the protein bonds [32]. Ghoddushi [33] reported that the starter culture used in cheese production was active on cohesiveness values. Yerlikaya [34] in his research on the production of cheese with capers found that the cohesiveness values of the cheese were 0.44–0.68 at the beginning of the storage and 0.15–0.29 at the end of the storage. Şener [24] found the cohesiveness values of the samples in the range of 0.21–0.56 in the research using transglutaminase enzyme in cheese production. In a study by Cankurt [7], it was stated that the cohesiveness values of the cheese were in the range of 0.77–0.89. The cohesiveness value in our study was found to be lower than those reported in the literature. It is considered that this can be caused by the high concentration of salt in cheese (Table 7).

Chewiness is a not characteristic of cheese that is directly defined, but rather it is calculated using multiple variables [35]. Hardness, cohesiveness, and springiness values are used in the calculation of chewiness [24]. The higher the chewiness value of cheese, the more force is needed to chew the sample, and the chewiness value can be expressed as non-chewability (Table 8).

It is clear that the stabilizers are significantly useful on the chewiness value. According to the benefits of the first day, the chewiness values of the samples can be said to be close to each other. Although there was an increase in all the examples, there was a continuous decline in both control samples. The control sample showed the highest decline among the cheeses with brine containing 8% salt. The cheeses with the highest mean were the cheeses kept in brine with gelatin. In cheeses with brine containing 12% salt, the lowest value was 112.91 g in cheese samples with brine fortified with 0.50% guar gum, while the highest value was 0.75% in the case of brine with 0.75% guar gum. When all data were examined, no correlation was found between the use of stabilizer and chewiness values except for the continuous decrease of the control sample. When the chewiness values of white cheese were examined, different results were obtained. Şener [24] investigated the effect of using different enzymes on the texture of cheese and found that chewiness values in the control cheeses were 180 at the beginning of storage and 90 at the end of storage. Cankurt [7] reported that the chewiness values in the cheese samples with hydrosol fluctuated throughout the storage and showed a significant decline at the end of storage. The chewiness results in our study are similar to results showed by Cankurt [7].

Adhesiveness can be defined as a force, which allows the elimination of the force of attraction between food and surfaces such as the palate, tongue, or teeth during the consumption of food [36]. Adhesiveness values were very close to each other on the first day of storage and followed a fluctuating course according to storage days (Table 9). No meaningful interpretation and comparison could be made due to this fluctuation.

Şener [24] stated that the value of adhesiveness in the cheeses produced decreased at the end of the storage. Cankurt [7] noted that the adhesiveness values of the cheeses produced with hydrosol were between −3.19 and −4.81 g.sn at the beginning of the storage and between −2.70 and −3.36 g.sn at the end of storage.

Gumminess can be expressed as the braking force needed to prepare the semi-solid food for ingestion [35]. It is also defined as the value found by multiplication of hardness and cohesiveness [34]. The gumminess values of our samples were close to each other on the first day, like other parameters (Table 10). In all cheese samples, it was observed that the gumminess value increased from 1 to 15 days and then decreased again. The lowest value in samples with brine containing 8% salt was recorded in the control sample while the highest values were obtained in samples kept in brine with gelatin. Among the cheese samples with brine containing 12% salt, the highest values were also obtained in those in brine with gelatin. Accordingly, the use of gelatin can be considered to increase the gumminess of cheeses. Cankurt [7], measuring the gumminess value of cheeses produced with hydrosol, reported the highest level of 282.03 g for the control sample and the lowest level of 191.9 g for the cheese sample with garlic hydrosol on the first day, while at the end of storage, the highest value was in the sample with garlic hydrosol as 223.1 g and the lowest value was in the control sample as 107.9 g.

Resilience values were similar to each other on the first day. No serious decline was observed during the first fifteen days, but at the end of storage, the resilience values of the samples decreased by more than half (Table 11).

In the samples with brine containing 8% salt, the lowest value was recorded in the control sample, while the highest values were among the samples with brine containing 12% salt. The linear relationship between resilience values and cohesiveness values is remarkable. Similar changes are observed when the values of these two parameters are compared. Cankurt [7] reported that the resilience values of the cheeses produced with hydrosol increased until the 60th day in general and then decreased again, and that the first day values were between 0.39–0.45 g and the last day between 0.44–0.48 g. When the resilience results of our study are examined, a decline is observed towards the end of storage. 

Springiness can be defined as the degree of being able to return to its previous state after the removal of the deformation force applied on an item [24,37,38]. At the end of storage, all of the samples with brine containing 8% salt had a decrease in springiness values. In general, springiness values tended to increase first and then decrease again. On the first day, it was observed that the springiest samples were control samples. At the end of storage, it was the control sample which lost the most springiness among the samples with brine containing 8% salt. Among the samples with brine containing 12% salt, the highest loss was observed in the sample with 1% gelatin (Table 12).

There is an inverse correlation between the rate of proteolysis and springiness in cheese during the ripening phase [33]. In a study by Yerlikaya [34] on cheese production with capers, the value of springiness was found as 0.87–0.94 on the first day of storage, and it was between 0.80–0.87 at the end of 90 days of storage. Cankurt [7] reported that the springiness values of the cheeses produced with hydrosol were between 0.92 and 1.

### 3.4. Sensorial Properties

Samples were scored by an average of 15 panelists during the first day and storage. The panelists evaluated the appearance, texture, taste, and general acceptance of the samples. When the samples were assessed in terms of appearance, they received very close scores on the first day, and there was no statistical difference among them (Table 13). During storage, there were a constant decline in appearance values. This was because the products softened towards the end of storage.

In general, the samples received high scores for their appearance until the end of storage. However, some cheeses did not comply with this trend. These are the samples with brine containing 8% salt and 0.25% guar gum and the control sample. Samples that were kept in brine containing 12% salt and could not maintain their appearance at a high level were samples with 0.5% guar gum and control samples. In other words, in both cases, the control sample was among the examples that received the lowest score in terms of appearance. 

When the texture values are taken into consideration, it is seen that cheeses stored in brine containing 8% salt scored the lower degrees. The real problem was manifested in samples kept in brine with guar and in control sample. In samples kept in brine with guar gum, the scores given to the texture decreased as the gum ratio decreased. In the cheeses with brine containing 12% salt, the lowest score was received by the control sample and after the example with guar gum (Table 14).

Taste scores are also parallel to texture scores. The taste of the samples decreased with storage. Again, the lowest scoring problematic samples in terms of taste were the cheese kept in brine with guar gum and the control cheese. The sample kept in brine 3% gelatin received a relatively lower score compared to other samples in terms of taste at the end of one-month storage. Scores given to the samples with brine containing 12% salt were lower. This was because samples were characterized as salty by panelists. Apart from salinity, strange taste and smell were not reported. There was only a slight unfamiliar taste and odor that could not be described in the sample with 3% gelatin (Table 15).

When all the sensory characteristics considered as general acceptance were examined the lower values were received by samples with brine containing 8% salt and 0.25% or 0.5% guar gum and control samples. Samples with brine containing 8% and 12% salt received the same score at the end of storage. The most-liked samples were those with gelatin and carrageenan (Table 16).

## 4. Conclusions

Since cheese in industrial production is kept in brine containing 12% salt, it is incredibly salty and causes consumer rejection. In addition, considering the adverse effects of excessive salt on health, the necessity to develop an alternative method for brine salting has emerged. Besides, by adding a stabilizer to the brine of the cheese, which is kept in its packaging, cheese is prevented from softening by absorbing water from brine. In other words, it ensures the preservation of the unique texture of the cheese. Cheese, which absorbs water from brine, also adds its water to brine, and thus brine is clouded. When cheeses are kept in brine with high salt content, they become extraordinarily salty, and their unique flavor is not perceived. Only the taste of salt is seen. Besides, because of the excessive salt, the starter culture that creates the aroma is suppressed, and the desired flavor and smell do not form in the cheese.

Furthermore, while cheese waits in brine, some food ingredients pass into the brine. Brine was jellified to prevent the passage of these ingredients into the brine. When cheeses are placed in brine, they float due to the density of brine. The remaining portion of cheese on the surface is dehydrated and become mold-infested in a few days. This problem does not occur in cheese that is kept in brine with stabilizer. By jellifying the brine, the cheese is prevented from floating and growing mold. At the same time, it is also intended to apply this to products such as olives and pickles kept in brine. In conclusion, adding of different stabilizers to cheese brine did not negatively affect the textural and sensory properties of the cheese, and also the cheese kept in brine with 8% salt content lasted for 30 days without softening. Among the stabilizers, guar gum did not provide the desired effect.

## Figures and Tables

**Table 1:** Dry Matter (g/100) of cheese samples during storage.

8%Brine				12%Brine		
Samples	1st Day	15th Day	30th Day	1st Day	15th Day	30th Day
%0.5 C	39.04 ± 0.29 ^Ca^	37.77 ± 0.36 ^BCa^	39.17 ± 0.33 ^Aa^	39.72 ± 0.23 ^BCb^	39.54 ± 0.13 ^ABa^	38.35 ± 0.22 ^Aa^
%1.0 C	41.81 ± 0.66 ^ABCb^	36.94 ± 0.15 ^Cb^	39.59 ± 0.41 ^Aa^	42.51 ± 0.25 ^ABa^	39.64 ± 0.28 ^Aa^	39.76 ± 0.09 ^Aa^
%1.5 C	42.03 ± 0.65 ^ABCa^	38.59 ± 0.13 ^ABCa^	39.79 ± 0.66 ^Aa^	44.74 ± 1.09 ^Aa^	39.90 ± 0.58 ^Aa^	40.19 ± 0.44 ^Aa^
%0.25 G	37.97 ± 0.42 ^Ba^	34.33 ± 0.14 ^Db^	34.39 ± 0.27 ^Cb^	39.10 ± 0.72 ^ABb^	38.53 ± 0.48 ^ABa^	39.31 ± 0.31 ^ABa^
%0.50 G	38.41 ± 0.26 ^Ba^	35.97 ± 0.37 ^CDa^	38.50 ± 0.12 ^Ba^	41.86 ± 0.77 ^Aa^	38.47 ± 0.54 ^ABa^	40.33 ± 0.16 ^Aa^
%0.75 G	37.81 ± 0.22 ^Ba^	37.63 ± 0.34 ^BCa^	38.88 ± 0.19 ^ABa^	40.66 ± 0.5 ^ABa^	40.02 ± 0.43 ^Aa^	40.43 ± 0.52 ^Aa^
%0.25 X	39.21 ± 0.32 ^Aa^	36.30 ± 0.16 ^Ca^	38.39 ± 0.1 ^ABCa^	40.89 ± 0.5 ^Aa^	38.91 ± 0.59 ^ABa^	37.29 ± 0.31 ^Cb^
%0.50 X	39.11 ± 0.16 ^Ab^	36.97 ± 0.13 ^BCb^	39.16 ± 0.2 ^ABa^	40.89 ± 0.34 ^Aa^	39.66 ± 0.43 ^Aa^	38.14 ± 0.14 ^BCb^
%0.75 X	39.47 ± 0.22 ^Aa^	37.68 ± 0.75 ^ABCa^	39.14 ± 0.46 ^ABa^	40.65 ± 0.33 ^Aa^	39.86 ± 0.15 ^Aa^	39.85 ± 0.23 ^Aa^
%1 J	39.33 ± 0.77 ^BCa^	34.75 ± 0.26 ^Cb^	35.15 ± 0.07 ^Cb^	41.24 ± 0.46 ^ABCa^	38.56 ± 0.48 ^Ba^	39.06 ± 0.5 ^ABa^
%2 J	38.52 ± 0.28 ^Ca^	37.04 ± 0.46 ^Ba^	35.27 ± 0.15 ^Cb^	42.18 ± 0.26 ^ABa^	41.44 ± 0.17 ^Ab^	40.10 ± 0.23 ^Aa^
%3 J	40.31 ± 0.49 ^BCa^	38.55 ± 0.16 ^Ba^	37.83 ± 0.12 ^Ba^	43.84 ± 0.95 ^Aa^	42.17 ± 0.28 ^Aa^	40.54 ± 0.71 ^Aa^
K	39.06 ± 0.27 ^Ab^	33.59 ± 0.09 ^Bb^	35.15 ± 0.22 ^Ab^	39.12 ± 0.46 ^Ab^	36.94 ± 0.37 ^Ab^	35.18 ± 0.41 ^Ab^

C: Carrageenan G: Guar gum X: Xanthan gum J: Gelatin K: Control. A–D statically shows the difference between samples, a–c shows the difference between days.

**Table 2:** pH properties of cheese samples during storage.

8%Brine				12%Brine		
Samples	1st Day	15th Day	30th Day	1st Day	15th Day	30th Day
%0.5 C	4.82 ± 0.02 ^Bb^	4.82 ± 0.01 ^Bb^	4.54 ± 0.01 ^Bb^	4.90 ± 0.02 ^Aa^	4.93 ± 0.01 ^Ab^	4.85 ± 0.01 ^Ab^
%1.0 C	4.81 ± 0.01 ^Bb^	4.79 ± 0.01 ^Bb^	4.53 ± 0.01 ^Bb^	4.82 ± 0.01 ^Bb^	4.95 ± 0.01 ^Aa^	4.86 ± 0.01 ^Ab^
%1.5 C	4.82 ± 0.01 ^Bb^	4.81 ± 0.01 ^Bb^	4.55 ± 0.01 ^Bb^	4.88 ± 0.01 ^ABa^	4.94 ± 0.01 ^Aa^	4.83 ± 0.01 ^Ab^
%0.25 G	4.90 ± 0.01 ^Aa^	4.94 ± 0.01 ^ABa^	4.72 ± 0.01 ^Ca^	4.92 ± 0.02 ^Aa^	5.00 ± 0.02 ^Aa^	4.82 ± 0.01 ^Bb^
%0.50 G	4.94 ± 0.01 ^Aa^	4.81 ± 0.01 ^Cb^	4.58 ± 0.01 ^Da^	4.91 ± 0.01 ^Aa^	5.00 ± 0.01 ^Aa^	4.88 ± 0.01 ^Aa^
%0.75 G	4.90 ± 0.05 ^Aa^	4.89 ± 0.01 ^Ba^	4.61 ± 0.01 ^Dab^	4.92 ± 0.01 ^Aa^	5.00 ± 0.01 ^Aa^	4.80 ± 0.00 ^Bb^
%0.25 X	4.88 ± 0.01 ^Aa^	4.92 ± 0.01 ^Aa^	4.60 ± 0.01 ^Ca^	4.86 ± 0.01 ^Aa^	4.97 ± 0.01 ^Aa^	4.82 ± 0.01 ^Ab^
%0.50 X	4.91 ± 0.01 ^Aa^	4.92 ± 0.01 ^Aa^	4.57 ± 0.01 ^Cb^	4.87 ± 0.01 ^Aa^	4.98 ± 0.02 ^Aa^	4.82 ± 0.01 ^Ab^
%0.75 X	4.90 ± 0.01 ^Aa^	4.93 ± 0.00 ^Aa^	4.68 ± 0.02 ^Ba^	4.87 ± 0.02 ^Aa^	4.98 ± 0.02 ^Aa^	4.88 ± 0.01 ^Aa^
%1 J	4.90 ± 0.01 ^ABa^	4.94 ± 0.01 ^BCa^	4.83 ± 0.01 ^Ca^	4.86 ± 0.01 ^Aa^	4.99 ± 0.00 ^ABa^	4.97 ± 0.01 ^Aa^
%2 J	4.93 ± 0.01 ^Aa^	4.92 ± 0.01 ^Ca^	4.90 ± 0.02 ^Ba^	4.89 ± 0.01 ^ABa^	5.02 ± 0.01 ^Aa^	4.97 ± 0.01 ^Aa^
%3 J	4.91 ± 0.01 ^ABa^	4.94 ± 0.01 ^BCa^	4.91 ± 0.01 ^ABa^	4.87 ± 0.01 ^Ba^	4.98 ± 0.01 ^ABa^	4.95 ± 0.01 ^ABa^
K	4.91 ± 0.01 ^Aa^	4.90 ± 0.00 ^Ba^	4.63 ± 0.03 ^Ab^	4.92 ± 0.00 ^Aa^	5.03 ± 0.01 ^Aa^	4.70 ± 0.01 ^Ab^

C: Carrageenan G: Guar gum X: Xanthan gum J: Gelatin K: Control. A–D statically shows the difference between samples, a–b shows the difference between days.

**Table 3:** Salt content (%) of cheese samples during storage.

8%Brine				12%Brine		
Samples	1st Day	15th Day	30th Day	1st Day	15th Day	30th Day
%0.5 C	3.45 ± 0.13 ^Ba^	3.67 ± 0.08 ^Bb^	4.32 ± 0.07 ^Ba^	4.27 ± 0.05 ^Aab^	5.01 ± 0.03 ^Ab^	5.84 ± 0.05 ^Aa^
%1.0 C	3.29 ± 0.04 ^Bab^	3.60 ± 0.03 ^Bb^	4.26 ± 0.03 ^Ba^	4.13 ± 0.0 5^Ab^	4.90 ± 0.08 ^Ab^	5.78 ± 0.36 ^Aa^
%1.5 C	3.16 ± 0.06 ^Bb^	3.50 ± 0.04 ^Bb^	4.12 ± 0.06 ^Ba^	4.02 ± 0.03 ^Ab^	4.77 ± 0.04 ^Ab^	5.72 ± 0.03 ^Aa^
%0.25 G	3.80 ± 0.05 ^Ba^	4.14 ± 0.04 ^Ba^	4.26 ± 0.04 ^Ca^	4.54 ± 0.09 ^Aa^	5.55 ± 0.04 ^Aa^	6.00 ± 0.04 ^Aa^
%0.50 G	3.44 ± 0.02 ^BCa^	4.02 ± 0.03 ^Ba^	4.04 ± 0.03 ^Db^	4.49 ± 0.05 ^Aa^	5.45 ± 0.11 ^Aa^	5.94 ± 0.03 ^Aa^
%0.75 G	3.33 ± 0.05 ^Cb^	4.02 ± 0.04 ^Ba^	4.29 ± 0.04 ^Ca^	4.43 ± 0.12 ^Aa^	5.43 ± 0.12 ^Aa^	5.59 ± 0.04 ^Bb^
%0.25 X	3.75 ± 0.02 ^Ca^	4.07 ± 0.05 ^Ba^	4.23 ± 0.06 ^Ca^	4.50 ± 0.07 ^ABa^	5.30 ± 0.03 ^Aa^	5.71 ± 0.03 ^Ba^
%0.50 X	3.80 ± 0.06 ^Ca^	4.21 ± 0.03 ^Ba^	4.23 ± 0.03 ^Ca^	4.62 ± 0.06 ^Aa^	5.42 ± 0.08 ^Aa^	5.81 ± 0.02 ^ABa^
%0.75 X	3.69 ± 0.05 ^Ca^	4.05 ± 0.04 ^Ba^	4.26 ± 0.03 ^Ca^	4.20 ± 0.01 ^Bb^	5.25 ± 0.04 ^Aa^	5.91 ± 0.02 ^Aa^
%1 J	3.74 ± 0.04 ^Ba^	4.00 ± 0.1 ^Ba^	4.05 ± 0.05 ^Bb^	4.59 ± 0.07 ^Aa^	5.14 ± 0.04 ^Ab^	5.78 ± 0.05 ^Ab^
%2 J	3.26 ± 0.02 ^Cc^	3.87 ± 0.1 ^Bb^	4.23 ± 0.05 ^Ba^	4.29 ± 0.07 ^Ab^	5.10 ± 0.02 ^Ab^	5.92 ± 0.03 ^Aa^
%3 J	3.04 ± 0.12 ^Cc^	3.61 ± 0.1 ^Bb^	4.03 ± 0.02 ^Bb^	4.23 ± 0.03 ^Ab^	5.04 ± 0.06 ^Ab^	5.80 ± 0.02 ^Aa^
K	3.67 ± 0.05 ^Ba^	4.13 ± 0.03 ^Ba^	4.01 ± 0.03 ^Bb^	4.60 ± 0.11 ^Aa^	5.31 ± 0.1 ^Aa^	5.40 ± 0.04 ^Ab^

C: Carrageenan G: Guar gum X: Xanthan gum J: Gelatin K: Control. A–B statically shows the difference between samples, a–b shows the difference between days.

**Table 4:** TMAB (total mesophilic aerobic bacteria) count (log CFU/g) of cheese samples during storage.

8%Brine				12%Brine		
Samples	1st Day	15th Day	30th Day	1st Day	15th Day	30th Day
%0.5 C	8.46 ± 0.18 ^Ab^	9.18 ± 0.07 ^Aab^	9.50 ± 0.17 ^Aa^	8.85 ± 0.20 ^Aa^	9.15 ± 0.17 ^Aa^	9.37 ± 0.17 ^Aa^
%1.0 C	8.45 ± 0.23 ^Aa^	9.32 ± 0.25 ^Aa^	9.55 ± 0.12 ^Aa^	9.07 ± 0.23 ^Aa^	9.36 ± 0.16 ^Aa^	9.54 ± 0.18 ^Aa^
%1.5 C	8.88 ± 0.10 ^Ab^	9.87 ± 0.05 ^Aa^	9.95 ± 0.11 ^Aa^	8.66 ± 0.18 ^Aa^	8.96 ± 0.20 ^Aa^	9.19 ± 0.15 ^Aa^
%0.25 G	8.69 ± 0.14 ^Ab^	9.44 ± 0.14 ^Aab^	9.74 ± 0.16 ^Aa^	8.30 ± 0.13 ^Aa^	8.58 ± 0.17 ^Ba^	8.79 ± 0.14 ^Ba^
%0.50 G	8.18 ± 0.10 ^Ab^	8.86 ± 0.11 ^ABa^	9.21 ± 0.07 ^ABa^	8.37 ± 0.20 ^Aa^	8.61 ± 0.18 ^Ba^	8.89 ± 0.12 ^Ba^
%0.75 G	8.31 ± 0.16 ^Ab^	9.07 ± 0.08 ^ABa^	9.36 ± 0.13 ^ABa^	8.62 ± 0.16 ^Aa^	8.94 ± 0.12 ^ABa^	9.16 ± 0.01 ^ABa^
%0.25 X	8.60 ± 0.10 ^Ab^	9.38 ± 0.13 ^Aa^	9.65 ± 0.08 ^Aa^	8.53 ± 0.09 ^Aa^	8.84 ± 0.16 ^Aa^	9.10 ± 0.09 ^Aa^
%0.50 X	8.59 ± 0.14 ^Ab^	9.28 ± 0.03 ^Aa^	9.67 ± 0.09 ^Aa^	8.65 ± 0.20 ^Aa^	8.97 ± 0.21 ^Aa^	9.16 ± 0.17 ^Aa^
%0.75 X	8.60 ± 0.11 ^Ab^	9.36 ± 0.09 ^Aa^	9.65 ± 0.09 ^Aa^	8.43 ± 0.25 ^Aa^	8.77 ± 0.22 ^Aa^	8.97 ± 0.19 ^Aa^
%1 J	8.12 ± 0.23 ^Aa^	8.99 ± 0.18 ^ABa^	9.18 ± 0.17 ^Aa^	8.34 ± 0.07 ^Aa^	8.65 ± 0.11 ^Ba^	8.86 ± 0.10 ^Aa^
%2 J	7.95 ± 0.33 ^Aa^	8.87 ± 0.15 ^ABa^	8.97 ± 0.24 ^Aa^	8.45 ± 0.05 ^Aa^	8.74 ± 0.08 ^ABa^	8.98 ± 0.03 ^Aa^
%3 J	8.63 ± 0.17 ^Ab^	9.56 ± 0.13 ^Aab^	9.66 ± 0.20 ^Aa^	8.18 ± 0.14 ^Aa^	8.49 ± 0.24 ^Ba^	8.75 ± 0.16 ^Aa^
K	8.04 ± 0.08 ^Ab^	8.84 ± 0.09 ^Aa^	9.06 ± 0.09 ^Aa^	8.14 ± 0.15 ^Aa^	8.45 ± 1.44 ^Aa^	8.63 ± 1.50 ^Aa^

C: Carrageenan G: Guar gum X: Xanthan gum J: Gelatin K: Control. A–B statically shows the difference between samples, a–b shows the difference between days.

**Table 5:** LAB count (log CFU/g) of cheese samples during storage.

8%Brine				12%Brine		
Samples	1st Day	15th Day	30th Day	1st Day	15th Day	30th Day
%0.5 C	8.29 ± 0.21 ^Aa^	8.66 ± 0.17 ^Aa^	8.81 ± 0.16 ^Aa^	8.37 ± 0.13 ^Aa^	8.73 ± 0.14 ^Aa^	8.96 ± 0.14 ^Aa^
%1.0 C	8.25 ± 0.18 ^Aa^	8.70 ± 0.15 ^Aa^	8.88 ± 0.20 ^Aa^	8.49 ± 0.12 ^Aa^	8.87 ± 0.12 ^Aa^	9.09 ± 0.13 ^Aa^
%1.5 C	7.97 ± 0.41 ^Aa^	8.42 ± 0.31 ^Aa^	8.60 ± 0.25 ^Aa^	8.25 ± 0.21 ^Aa^	8.62 ± 0.22 ^Aa^	8.84 ± 0.23 ^Aa^
%0.25 G	8.18 ± 0.25 ^Aa^	8.60 ± 0.21 ^Aa^	8.77 ± 0.21 ^Aa^	7.94 ± 0.16 ^Aa^	8.30 ± 0.17 ^Aa^	8.50 ± 0.17 ^Aa^
%0.50 G	7.98 ± 0.20 ^Aa^	8.44 ± 0.17 ^Aa^	7.87 ± 1.48 ^Aa^	7.84 ± 0.15 ^Aa^	8.20 ± 0.15 ^Aa^	8.40 ± 0.16 ^Aa^
%0.75 G	8.01 ± 0.10 ^Ab^	8.46 ± 0.07 ^Aab^	8.60 ± 0.07 ^Aa^	8.25 ± 0.11 ^Aa^	8.62 ± 0.12 ^Aa^	8.83 ± 0.12 ^Aa^
%0.25 X	8.25 ± 0.15 ^Aa^	8.64 ± 0.13 ^Aa^	8.90 ± 0.06 ^Aa^	8.22 ± 0.18 ^Aa^	8.59 ± 0.19 ^Aa^	8.81 ± 0.20 ^Aa^
%0.50 X	8.40 ± 0.16 ^Aa^	8.84 ± 0.16 ^Aa^	9.02 ± 0.09 ^Aa^	8.30 ± 0.25 ^Aa^	8.67 ± 0.26 ^Aa^	8.89 ± 0.26 ^Aa^
%0.75 X	8.13 ± 0.20 ^Aa^	8.55 ± 0.15 ^Aa^	8.72 ± 0.13 ^Aa^	7.91 ± 0.22 ^Aa^	8.26 ± 0.23 ^Aa^	8.47 ± 0.23 ^Aa^
%1 J	8.05 ± 0.16 ^Aa^	8.45 ± 0.18 ^Aa^	8.61 ± 0.11 ^Aa^	7.55 ± 0.21 ^Aa^	7.89 ± 0.22 ^Aa^	8.09 ± 0.23 ^Aa^
%2 J	7.73 ± 0.23 ^Aa^	8.12 ± 0.17 ^Aa^	8.29 ± 0.10 ^Aa^	7.99 ± 0.12 ^Aa^	8.35 ± 0.12 ^Aa^	8.56 ± 0.13 ^Aa^
%3 J	7.65 ± 0.21 ^Aa^	8.04 ± 0.12 ^Aa^	8.20 ± 0.06 ^Aa^	7.89 ± 0.31 ^Aa^	8.25 ± 0.32 ^Aa^	8.45 ± 0.33 ^Aa^
K	7.73 ± 0.19 ^Aa^	8.12 ± 0.09 ^Aa^	8.28 ± 0.10 ^Aa^	7.99 ± 0.12 ^Aa^	8.34 ± 0.13 ^Aa^	8.55 ± 0.13 ^Aa^

C: Carrageenan G: Guar gum X: Xanthan gum J: Gelatin K: Control. A statically shows the difference between samples, a–b shows the difference between days.

**Table 6:** Hardness (g) of cheese samples during storage.

8%Brine				12%Brine		
Samples	1st Day	15th Day	30th Day	1st Day	15th Day	30th Day
%0.5 C	228.2 ± 31.3 ^Cb^	566.1 ± 50.5 ^Aa^	382.1 ± 33.8 ^Aab^	374.0 ± 21.1 ^ABa^	596.27 ± 81.0 ^Aa^	707.52 ± 148.5 ^Aa^
%1.0 C	233.5 ± 28.3 ^BCb^	485.6 ± 35.3 ^Aa^	333.0 ± 54.2 ^Aab^	381.19 ± 21.5 ^Aa^	609.6 ± 71.7 ^Aa^	625.8 ± 39.0 ^Aa^
%1.5 C	235.5 ± 27.6 ^BCa^	381.5 ± 42.2 ^Aa^	376.6 ± 29.1 ^Aa^	387.65 ± 21.9 ^Aa^	672.6 ± 57.5 ^Aa^	774.8 ± 154.1 ^Aa^
%0.25 G	223.5 ± 30.7 ^Aa^	227.6 ± 36.6 ^Ba^	299.6 ± 49.7 ^Ca^	349.6 ± 19.7 ^Aa^	574.9 ± 27.5 ^Aa^	616.8 ± 82.1 ^ABa^
%0.50 G	225.3 ± 30.9 ^Aa^	385.6 ± 23.9 ^ABa^	388.5 ± 70.5 ^BCa^	354.3 ± 20.3 ^Ab^	486.3 ± 86.7 ^ABab^	684.6 ± 31.7 ^ABa^
%0.75 G	231.6 ± 26.8 ^Aa^	344.9 ± 40.9 ^ABa^	454.9 ± 46.0 ^BCa^	353.2 ± 19.9 ^Ab^	592.4 ± 76.5 ^Aab^	882.7 ± 66.8 ^Aa^
%0.25 X	223.4 ± 27.4 ^Ba^	331.9 ± 46.8 ^Ba^	304.7 ± 23.3 ^Aa^	367.2 ± 20.7 ^Aa^	543.5 ± 53.9 ^ABa^	495.7 ± 44.2 ^Aa^
%0.50 X	229.7 ± 20.1 ^Ba^	443.3 ± 60.6 ^Aba^	364.2 ± 24.5 ^Aa^	370.0 ± 20.9 ^Aa^	650.8 ± 71.6 ^Aa^	620.5 ± 130.3 ^Aa^
%0.75 X	235.7 ± 26.8 ^Ba^	503.0 ± 32.8 ^Aba^	445.7 ± 54.8 ^Aa^	374.3 ± 21.1 ^Aa^	650.4 ± 45.1 ^Aa^	651.2 ± 64.7 ^Aa^
%1 J	227.5 ± 31.2 ^Ca^	279.91 ± 47.1 ^Aa^	446.8 ± 48.0 ^Ba^	375.4 ± 21.2 ^ABb^	380.2 ± 18.3 ^Ab^	853.7 ± 65.2 ^ABa^
%2 J	231.7 ± 28.6 ^Ca^	422.7 ± 40.9 ^Aa^	440.84 ± 54.9 ^Ba^	378.6 ± 21.4 ^ABb^	672.0 ± 37.6 ^Ab^	1052.3 ± 82.8 ^Aa^
%3 J	237.1 ± 25.2 ^BCb^	449.9 ± 9.0 ^Aa^	556.8 ± 31.1 ^Ba^	383.3 ± 21.6 ^Aa^	649.4 ± 340.7 ^Aa^	1013.6 ± 134.4 ^Aa^
K	233.1 ± 31.4 ^Aa^	221.6 ± 30.4 ^Aa^	186.3 ± 21.9 ^Ba^	376.8 ± 12.7 ^Aa^	358.6 ± 20.2 ^Aa^	433.6 ± 39.9 ^Aa^

C: Carrageenan G: Guar gum X: Xanthan gum J: Gelatin K: Control. A–C statically shows the difference between samples, a–b shows the difference between days.

**Table 7:** Cohesiveness of cheese samples during storage.

8%Brine				12%Brine		
Samples	1st Day	15th Day	30th Day	1st Day	15th Day	30th Day
%0.5 C	0.85 ± 0.02 ^Aa^	0.80 ± 0.03 ^Aa^	0.53 ± 0.06 ^Ab^	0.85 ± 0.02 ^Aa^	0.84 ± 0.01 ^Aa^	0.35 ± 0.09 ^Ab^
%1.0 C	0.86 ± 0.02 ^Aa^	0.83 ± 0.02 ^Aa^	0.34 ± 0.04 ^Ab^	0.86 ± 0.02 ^Aa^	0.83 ± 0.02 ^Aa^	0.34 ± 0.13 ^Ab^
%1.5 C	0.86 ± 0.02 ^Aa^	0.84 ± 0.02 ^Aa^	0.42 ± 0.02 ^Ab^	0.88 ± 0.02 ^Aa^	0.84 ± 0.02 ^Aa^	0.23 ± 0.05 ^Ab^
%0.25 G	0.83 ± 0.02 ^Aa^	0.85 ± 0.02 ^Aa^	0.40 ± 0.10 ^Ab^	0.79 ± 0.02 ^Aa^	0.83 ± 0.02 ^Aa^	0.40 ± 0.12 ^Ab^
%0.50 G	0.84 ± 0.02 ^Aa^	0.84 ± 0.02 ^Aa^	0.39 ± 0.13 ^Aa^	0.80 ± 0.02 ^Aa^	0.85 ± 0.03 ^Aa^	0.27 ± 0.04 ^Ab^
%0.75 G	0.84 ± 0.02 ^Aa^	0.85 ± 0.02 ^Aa^	0.38 ± 0.06 ^Ab^	0.80 ± 0.02 ^Aa^	0.83 ± 0.01 ^Aa^	0.58 ± 0.05 ^Ab^
%0.25 X	0.85 ± 0.02 ^Aa^	0.84 ± 0.01 ^Aa^	0.44 ± 0.09 ^Ab^	0.83 ± 0.02 ^Aa^	0.81 ± 0.04 ^Aa^	0.33 ± 0.16 ^Aa^
%0.50 X	0.84 ± 0.03 ^Aa^	0.82 ± 0.02 ^Aa^	0.48 ± 0.19 ^Aa^	0.84 ± 0.02 ^Aa^	0.83 ± 0.05 ^Aa^	0.20 ± 0.02 ^Ab^
%0.75 X	0.85 ± 0.02 ^Aa^	0.82 ± 0.02 ^Aa^	0.36 ± 0.11 ^Ab^	0.85 ± 0.02 ^Aa^	0.79 ± 0.02 ^Aab^	0.27 ± 0.16 ^Ab^
%1 J	0.85 ± 0.02 ^Aa^	0.85 ± 0.02 ^Aa^	0.36 ± 0.06 ^Ab^	0.85 ± 0.02 ^Aa^	0.85 ± 0.01 ^Aa^	0.26 ± 0.04 ^Ab^
%2 J	0.85 ± 0.02 ^Aa^	0.85 ± 0.01 ^Aa^	0.50 ± 0.08 ^Ab^	0.86 ± 0.02 ^Aa^	0.84 ± 0.01 ^Aa^	0.34 ± 0.09 ^Ab^
%3 J	0.86 ± 0.02 ^Aa^	0.85 ± 0.02 ^Aab^	0.37 ± 0.14 ^Ab^	0.87 ± 0.02 ^Aa^	0.83 ± 0.02 ^Aa^	0.29 ± 0.03 ^Ab^
K	0.87 ± 0.02 ^Aa^	0.84 ± 0.02 ^Aa^	0.35 ± 0.06 ^Ab^	0.89 ± 0.02 ^Aa^	0.83 ± 0.02 ^Aa^	0.40 ± 0.18 ^Aa^

C: Carrageenan G: Guar gum X: Xanthan gum J: Gelatin K: Control. A statically shows the difference between samples, a–b shows the difference between days.

**Table 8:** Chewiness (g) of cheese samples during storage.

8%Brine				12%Brine		
Samples	1st Day	15th Day	30th Day	1st Day	15th Day	30th Day
%0.5 C	181.83 ± 28.7 ^Ab^	404.01 ± 32.0 ^Aa^	175.96 ± 21.3 ^Ab^	276.74 ± 31.5 ^Aa^	469.46 ± 52.34 ^Aa^	205.47 ± 64.26 ^Aa^
%1.0 C	183.25 ± 29.0 ^Ab^	378.09 ± 36.84 ^Aa^	90.43 ± 15.40 ^Ab^	282.05 ± 32.1 ^Aa^	467.72 ± 44.33 ^Aa^	182.18 ± 73.09 ^Aa^
%1.5 C	183.7 ± 29.1 ^Aab^	299.94 ± 27.92 ^Aa^	134.63 ± 9.78 ^Ab^	286.8 ± 32.7 ^Aab^	521.88 ± 43.44 ^Aa^	160.57 ± 53.80 ^Ab^
%0.25G	178.09 ± 28.2 ^Aa^	185.65 ± 25.48 ^Ba^	92.55 ± 17.46 ^Ba^	258.70 ± 29.5 ^Aa^	446.19 ± 23.33 ^Aa^	210.38 ± 81.1 ^ABa^
%0.50G	179.54 ± 28.4 ^Aa^	307.84 ± 31.8 ^ABa^	123.56 ± 55.5 ^Ba^	262.15 ± 29.9 ^Aa^	390.65 ± 79.8 ^ABa^	147.14 ± 21.5 ^Ba^
%0.75G	180.24 ± 28.5 ^Aa^	278.54 ± 23.1 ^ABa^	135.07 ± 28.9 ^Ba^	261.35 ± 29.8 ^Aa^	449.22 ± 54.7 ^Aa^	456.00 ± 26.9 ^Aa^
%0.25X	180.4 ± 28.5 ^Aab^	263.49 ± 38.0 ^Ba^	93.18 ± 11.6 ^Ab^	271.70 ± 31.0 ^Aa^	402.57 ± 46.9 ^ABa^	146.98 ± 70.2 ^Aa^
%0.50X	169.71 ± 26.8 ^Aa^	338.80 ± 47.1 ^ABa^	134.68 ± 62.8 ^Aa^	273.82 ± 31.2 ^Ab^	495.90 ± 28.0 ^Aa^	112.91 ± 37.8 ^Ab^
%0.75X	182.01 ± 28.8 ^Aa^	376.27 ± 31.4 ^ABa^	140.21 ± 64.5 ^Aa^	277.0 ± 31.6 ^Aab^	463.93 ± 28.5 ^ABa^	139.68 ± 65.7 ^Ab^
%1 J	181.30 ± 28.7 ^Aa^	227.45 ± 42.2 ^Aa^	134.07 ± 34.1 ^Aa^	277.80 ± 31.7 ^Aa^	305.33 ± 16.3 ^Aa^	174.18 ± 29.6 ^Aa^
%2 J	182.36 ± 28.8 ^Aa^	341.48 ± 28.0 ^Aa^	177.91 ± 43.7 ^Aa^	280.19 ± 31.9 ^Aa^	522.14 ± 20.4 ^Aa^	314.82 ± 81.8 ^Aa^
%3 J	183.42 ± 29.0 ^Aa^	362.05 ± 13.7 ^Aa^	165.27 ± 64.4 ^Aa^	283.64 ± 32.3 ^Aa^	500.32 ± 262.2 ^Aa^	258.16 ± 67.9 ^Aa^
K	185.36 ± 29.3 ^Aa^	176.54 ± 27.9 ^Aa^	47.40 ± 17.8 ^Aa^	291.87 ± 33.3 ^Aa^	265.33 ± 30.2 ^Aa^	154.85 ± 77.3 ^Aa^

C: Carrageenan G: Guar gum X: Xanthan gum J: Gelatin K: Control. A–B statically shows the difference between samples, a–b shows the difference between days.

**Table 9:** Adhesiveness (g.sn) cheese of samples during storage.

8%Brine				12%Brine		
Samples	1st Day	15th Day	30th Day	1st Day	15th Day	30th Day
%0.5 C	−2.77 ± 0.78 ^Aa^	−2.46 ± 1.03 ^Aa^	−8.74 ± 3.59 ^Aba^	−1.40 ± 0.80 ^Aa^	−1.66 ± 0.43 ^Aa^	−5.91 ± 2.70 ^Aba^
%1.0 C	−2.79 ± 0.79 ^Aa^	−1.93 ± 0.67 ^Aa^	−40.10 ± 12.71 ^Ba^	−1.42 ± 0.82 ^Aa^	−2.16 ± 0.51 ^Aa^	−4.71 ± 2.82 ^Aa^
%1.5 C	−2.80 ± 0.79 ^Aa^	−1.83 ± 1.13 ^Aa^	−10.02 ± 2.94 ^Aba^	−1.45 ± 0.83 ^Aa^	−2.13 ± 0.38 ^Aa^	−9.53 ± 6.05 ^Aba^
%0.25 G	−2.71 ± 0.77 ^Aa^	−3.17 ± 0.30 ^Aa^	−25.10 ± 31.36 ^Aa^	−1.31 ± 0.75 ^Aa^	−1.55 ± 0.39 ^Aa^	−7.81 ± 5.42 ^Aa^
%0.50 G	−2.73 ± 0.77 ^Aa^	−3.52 ± 0.16 ^Aa^	−12.68 ± 10.36 ^Aa^	−1.32 ± 0.76 ^Aa^	−2.48 ± 0.68 ^Aa^	−14.38 ± 6.42 ^Aa^
%0.75 G	−2.74 ± 0.78 ^Aa^	−2.55 ± 1.03 ^Aa^	−21.90 ± 6.05 ^Aa^	−1.32 ± 0.76 ^Aa^	−2.35 ± 1.08 ^Aa^	−6.28 ± 2.39 ^Aa^
%0.25 X	−2.75 ± 0.78 ^Aa^	−2.45 ± 0.63 ^Aa^	−22.50 ± 17.21 ^Aa^	−1.37 ± 0.79 ^Aa^	−1.87 ± 0.61 ^Aa^	−17.68 ± 13.06 ^Aa^
%0.50 X	−2.62 ± 0.75 ^Aa^	−2.35 ± 0.62 ^Aa^	−11.67 ± 9.26 ^Aa^	−1.38 ± 0.79 ^Aa^	−2.13 ± 0.83 ^Aa^	−19.99 ± 11.43 ^Aa^
%0.75 X	−2.77 ± 0.78 ^Aa^	−2.05 ± 0.82 ^Aa^	−18.57 ± 11.93 ^Aa^	−1.40 ± 0.80 ^Aa^	−1.79 ± 0.49 ^Aa^	−16.11 ± 12.04 ^Aa^
%1 J	−2.76 ± 0.78 ^Aa^	−2.91 ± 0.61 ^Aa^	−15.57 ± 8.02 ^Aa^	−1.40 ± 0.81 ^Aa^	−2.13 ± 0.94 ^Aa^	−13.64 ± 3.49 ^Aa^
%2 J	−2.78 ± 0.79 ^Aa^	−1.69 ± 0.55 ^Aa^	−19.77 ± 10.23 ^Aa^	−1.42 ± 0.81 ^Aa^	−1.93 ± 0.58 ^Aa^	−15.50 ± 12.56 ^Aa^
%3 J	−2.79 ± 0.79 ^Aa^	−1.63 ± 0.35 ^Aa^	−12.30 ± 7.65 ^Aa^	−1.43 ± 0.82 ^Aa^	−1.86 ± 0.64 ^Aa^	−37.11 ± 18.12 ^Aa^
K	−2.82 ± 0.80 ^Aa^	−2.69 ± 0.76 ^Aa^	−15.40 ± 9.51 ^Aa^	−1.47 ± 0.85 ^Aa^	−1.34 ± 0.77 ^Aa^	−10.50 ± 4.46 ^Aa^

C: Carrageenan G: Guar gum X: Xanthan gum J: Gelatin K: Control. A statically shows the difference between samples, a–b shows the difference between days.

**Table 10:** Gumminess (g) of cheese samples during storage.

8%Brine				12%Brine		
Samples	1st Day	15th Day	30th Day	1st Day	15th Day	30th Day
%0.5 C	192.64 ± 26.8 ^Bb^	450.46 ± 39.5 ^Aa^	203.10 ± 20.6 ^Ab^	310.3 ± 15.2 ^ABa^	498.3 ± 61.3 ^Aa^	248.0 ± 79.3 ^Aa^
%1.0 C	194.14 ± 27.0 ^Bb^	403.63 ± 35.3 ^Aa^	110.55 ± 16.7 ^Ab^	316.2 ± 15.5 ^ABa^	503.6 ± 53.0 ^Aa^	215.4 ± 89.4 ^Aa^
%1.5 C	194.7 ± 27.1 ^Bab^	319.5 ± 33.1 ^Aa^	158.1 ± 11.4 ^Ab^	321.6 ± 15.7 ^Aab^	563.09 ± 46.4 ^Aa^	180.23 ± 62.4 ^Ab^
%0.25G	188.67 ± 26.2 ^Aa^	192.68 ± 27.24 ^Ba^	118.81 ± 24.7 ^Ba^	290.07 ± 14.2 ^Aa^	476.72 ± 20.1 ^Aa^	247.3 ± 89.9 ^ABa^
%0.50G	190.21 ± 26.4 ^Aa^	325.0 ± 22.9 ^ABa^	153.83 ± 57.6 ^Ba^	293.94 ± 14.4 ^Aa^	414.9 ± 80.6 ^ABa^	181.68 ± 22.0 ^Ba^
%0.75G	190.96 ± 26.5 ^Aa^	291.6 ± 29.9 ^ABa^	171.81 ± 35.3 ^Ba^	293.05 ± 14.3 ^Aa^	488.97 ± 61.0 ^Aa^	507.20 ± 24.2 ^Aa^
%0.25X	191.1 ± 26.6 ^BCa^	279.26 ± 39.8 ^Ca^	134.2 ± 25.8 ^Aa^	304.6 ± 14.9 ^ABa^	440.3 ± 48.4 ^ABa^	167.25 ± 79.2 ^Aa^
%0.50X	182.38 ± 23.1 ^Ca^	364.73 ± 49.7 ^BCa^	175.3 ± 70.7 ^Aa^	307.0 ± 15.0 ^ABb^	537.76 ± 33.8 ^Aa^	124.66 ± 37.8 ^Ac^
%0.75X	192.8 ± 26.8 ^ABCa^	413.8 ± 33.6 ^ABCa^	164.8 ± 68.0 ^Aa^	310.6 ± 15.2 ^Aab^	513.30 ± 31.4 ^Aa^	169.51 ± 80.4 ^Ab^
%1 J	192.08 ± 26.74 ^Ba^	236.95 ± 42.2 ^Ca^	162.88 ± 38.2 ^Aa^	311.4 ± 15.2 ^ABa^	322.2 ± 17.0 ^BCa^	223.19 ± 44.3 ^Aa^
%2 J	193.20 ± 26.8 ^Ba^	359.0 ± 31.5 ^ABCa^	218.8 ± 45.3 ^Aa^	314.1 ± 15.4 ^ABa^	561.04 ± 28.9 ^Aa^	363.9 ± 109.9 ^Aa^
%3 J	194.32 ± 27.05 ^Ba^	381.2 ± 11.5 ^ABCa^	202.0 ± 75.1 ^Aa^	318.04 ± 15.59 ^Aa^	534.7 ± 79.2 ^ABa^	296.74 ± 70.61 ^Aa^
K	196.38 ± 27.34 ^Aa^	187.03 ± 26.03 ^Aa^	65.9817.35 ^Aa^	327.26 ± 16.04 ^Aa^	297.51 ± 14.58 ^Aa^	180.30 ± 87.49 ^Aa^

C: Carrageenan G: Guar gum X: Xanthan gum J: Gelatin K: Control. A–C statically shows the difference between samples, a–b shows the difference between days.

**Table 11:** Resilience (g) of cheese samples during storage.

8%Brine				12%Brine		
Samples	1st Day	15th Day	30th Day	1st Day	15th Day	30th Day
%0.5 C	0.51 ± 0.03 ^Aa^	0.43 ± 0.01 ^Aa^	0.24 ± 0.03 ^Ab^	0.51 ± 0.01 ^Aa^	0.46 ± 0.02 ^Aa^	0.18 ± 0.04 ^Ab^
%1.0 C	0.52 ± 0.03 ^Aa^	0.47 ± 0.01 ^Aa^	0.14 ± 0.02 ^Ab^	0.52 ± 0.01 ^Aa^	0.44 ± 0.03 ^Aa^	0.18 ± 0.06 ^Ab^
%1.5 C	0.52 ± 0.03 ^Aa^	0.47 ± 0.03 ^Aa^	0.20 ± 0.01 ^Ab^	0.53 ± 0.01 ^Aa^	0.46 ± 0.01 ^Aa^	0.14 ± 0.03 ^Ab^
%0.25G	0.50 ± 0.03 ^Aa^	0.51 ± 0.02 ^Aa^	0.20 ± 0.05 ^Ab^	0.48 ± 0.01 ^Aa^	0.45 ± 0.02 ^Aa^	0.19 ± 0.05 ^Ab^
%0.50G	0.51 ± 0.03 ^Aa^	0.46 ± 0.02 ^Aa^	0.18 ± 0.05 ^Ab^	0.49 ± 0.01 ^Aa^	0.48 ± 0.02 ^Aa^	0.13 ± 0.01 ^Ab^
%0.75G	0.51 ± 0.03 ^Aa^	0.48 ± 0.02 ^Aa^	0.16 ± 0.02 ^Ab^	0.49 ± 0.01 ^Aa^	0.48 ± 0.01 ^Aa^	0.25 ± 0.02 ^Ab^
%0.25X	0.51 ± 0.03 ^Aa^	0.48 ± 0.01 ^Aa^	0.21 ± 0.05 ^Ab^	0.50 ± 0.01 ^Aa^	0.47 ± 0.03 ^Aa^	0.15 ± 0.07 ^Ab^
%0.50X	0.51 ± 0.04 ^Aa^	0.46 ± 0.02 ^Aa^	0.22 ± 0.10 ^Ab^	0.51 ± 0.01 ^Aa^	0.46 ± 0.03 ^Aa^	0.10 ± 0.02 ^Ab^
%0.75X	0.52 ± 0.03 ^Aa^	0.47 ± 0.02 ^Aa^	0.17 ± 0.05 ^Ab^	0.51 ± 0.01 ^Aa^	0.43 ± 0.03 ^Aa^	0.13 ± 0.06 ^Ab^
%1 J	0.51 ± 0.03 ^Aa^	0.50 ± 0.02 ^Aa^	0.19 ± 0.04 ^Ab^	0.52 ± 0.01 ^Aa^	0.49 ± 0.01 ^Aa^	0.12 ± 0.02 ^Ab^
%2 J	0.52 ± 0.03 ^Aa^	0.50 ± 0.01 ^Aa^	0.24 ± 0.04 ^Ab^	0.52 ± 0.01 ^Aa^	0.45 ± 0.02 ^Aa^	0.16 ± 0.03 ^Ab^
%3 J	0.52 ± 0.03 ^Aa^	0.50 ± 0.02 ^Aa^	0.19 ± 0.04 ^Ab^	0.53 ± 0.01 ^Aa^	0.43 ± 0.02 ^Aa^	0.12 ± 0.02 ^Ab^
K	0.52 ± 0.04 ^Aa^	0.50 ± 0.03 ^Aa^	0.15 ± 0.03 ^Ab^	0.54 ± 0.01 ^Aa^	0.49 ± 0.01 ^Aa^	0.19 ± 0.07 ^Ab^

C: Carrageenan G: Guar gum X: Xanthan gum J: Gelatin K: Control. A statically shows the difference between samples, a–b shows the difference between days.

**Table 12:** Springiness of cheese samples during storage.

8%Brine				12%Brine		
Samples	1st Day	15th Day	30th Day	1st Day	15th Day	30th Day
%0.5 C	0.93 ± 0.04 ^Aa^	0.90 ± 0.02 ^Aa^	0.86 ± 0.02 ^Aa^	0.89 ± 0.06 ^Aa^	0.94 ± 0.02 ^Aa^	0.83 ± 0.04 ^Aa^
%1.0 C	0.94 ± 0.04 ^Aa^	0.94 ± 0.01 ^Aa^	0.82 ± 0.08 ^Aa^	0.91 ± 0.07 ^Aa^	0.93 ± 0.02 ^Aa^	0.85 ± 0.02 ^Aa^
%1.5 C	0.94 ± 0.04 ^Aa^	0.94 ± 0.02 ^Aa^	0.85 ± 0.02 ^Aa^	0.92 ± 0.07 ^Aa^	0.93 ± 0.01 ^Aa^	0.89 ± 0.03 ^Aa^
%0.25G	0.91 ± 0.04 ^Aa^	0.96 ± 0.01 ^Aa^	0.79 ± 0.09 ^Aa^	0.83 ± 0.06 ^Aa^	0.94 ± 0.02 ^Aa^	0.84 ± 0.02 ^Aa^
%0.50G	0.92 ± 0.04 ^Aa^	0.95 ± 0.04 ^Aa^	0.78 ± 0.06 ^Aa^	0.84 ± 0.06 ^Aa^	0.94 ± 0.02 ^Aa^	0.81 ± 0.04 ^Aa^
%0.75G	0.92 ± 0.04 ^Aab^	0.96 ± 0.02 ^Aa^	0.79 ± 0.02 ^Ab^	0.84 ± 0.06 ^Aa^	0.92 ± 0.02 ^Aa^	0.90 ± 0.02 ^Aa^
%0.25X	0.92 ± 0.04 ^Aa^	0.94 ± 0.01 ^Aa^	0.71 ± 0.10 ^Aa^	0.87 ± 0.06 ^Aa^	0.91 ± 0.02 ^Aa^	0.88 ± 0.02 ^Aa^
%0.50X	0.92 ± 0.04 ^Aa^	0.93 ± 0.01 ^Aa^	0.78 ± 0.15 ^Aa^	0.88 ± 0.06 ^Aa^	0.92 ± 0.03 ^Aa^	0.90 ± 0.06 ^Aa^
%0.75X	0.93 ± 0.04 ^Aa^	0.91 ± 0.01 ^Aa^	0.84 ± 0.07 ^Aa^	0.89 ± 0.06 ^Aa^	0.90 ± 0.01 ^Aa^	0.83 ± 0.04 ^Aa^
%1 J	0.93 ± 0.04 ^Aa^	0.96 ± 0.01 ^Aa^	0.82 ± 0.02 ^Aa^	0.89 ± 0.07 ^Aa^	0.95 ± 0.01 ^Aa^	0.79 ± 0.04 ^Aa^
%2 J	0.93 ± 0.04 ^Aa^	0.95 ± 0.01 ^Aa^	0.81 ± 0.03 ^Aa^	0.90 ± 0.07 ^Aa^	0.93 ± 0.02 ^Aa^	0.88 ± 0.06 ^Aa^
%3 J	0.94 ± 0.04 ^Aa^	0.95 ± 0.02 ^Aa^	0.81 ± 0.05 ^Aa^	0.91 ± 0.07 ^Aa^	0.93 ± 0.03 ^Aa^	0.87 ± 0.09 ^Aa^
K	0.95 ± 0.04 ^Aa^	0.94 ± 0.04 ^Aa^	0.70 ± 0.09 ^Aa^	0.92 ± 0.06 ^Aa^	0.89 ± 0.06 ^Aa^	0.85 ± 0.03 ^Aa^

C: Carrageenan G: Guar gum X: Xanthan gum J: Gelatin K: Control. A statically shows the difference between samples, a–b shows the difference between days

**Table 13:** Appearance score of cheese samples during storage.

8%Brine				12%Brine		
Samples	1st Day	15th Day	30th Day	1st Day	15th Day	30th Day
%0.5 C	8.20 ± 0.27 ^Aa^	8.10 ± 0.22 ^Aa^	7.90 ± 0.22 ^Aa^	8.46 ± 0.29 ^Aa^	8.30 ± 0.45 ^Ab^	8.20 ± 0.27 ^Aa^
%1.0 C	8.40 ± 0.31 ^Aa^	8.34 ± 0.32 ^Aa^	8.10 ± 0.22 ^Aa^	8.80 ± 0.31 ^Aa^	8.80 ± 0.45 ^Aa^	8.50 ± 0.35 ^Aa^
%1.5 C	8.44 ± 0.43 ^Aa^	8.44 ± 0.43 ^Aa^	8.30 ± 0.27 ^Aa^	8.60 ± 0.42 ^Aa^	8.60 ± 0.55 ^Aa^	8.40 ± 0.42 ^Aa^
%0.25G	5.90 ± 0.82 ^Bb^	5.90 ± 1.02 ^Bb^	5.70 ± 0.76 ^Bc^	8.90 ± 0.22 ^Aa^	8.80 ± 0.27 ^Aa^	8.20 ± 0.27 ^Aa^
%0.50G	8.10 ± 0.42 ^ABa^	8.00 ± 0.00 ^ABa^	7.80 ± 0.27 ^ABb^	8.00 ± 0.00 ^ABb^	7.94 ± 0.13 ^ABb^	7.90 ± 0.22 ^ABb^
%0.75G	8.20 ± 0.27 ^ABa^	8.16 ± 0.23 ^ABa^	8.10 ± 0.22 ^Aa^	8.80 ± 0.27 ^Aa^	8.70 ± 0.45 ^Aa^	8.50 ± 0.35 ^Aa^
%0.25X	8.84 ± 0.23 ^Aa^	8.80 ± 0.45 ^Aa^	8.60 ± 0.42 ^Aa^	8.86 ± 0.22 ^Aa^	9.00 ± 0.00 ^Aa^	8.80 ± 0.27 ^Aa^
%0.50X	8.76 ± 0.25 ^Aa^	8.80 ± 0.45 ^Aa^	8.60 ± 0.42 ^Aa^	8.86 ± 0.22 ^Aa^	9.00 ± 0.00 ^Aa^	8.80 ± 0.27 ^Aa^
%0.75X	8.30 ± 0.27 ^Aaa^	8.30 ± 0.45 ^Aa^	8.10 ± 0.22 ^Aa^	9.00 ± 0.00 ^Aba^	9.00 ± 0.00 ^Aa^	8.90 ± 0.22 ^Aa^
%1 J	8.00 ± 0.35 ^Aa^	7.80 ± 0.45 ^Aa^	7.70 ± 0.45 ^Ab^	9.00 ± 0.00 ^Aba^	9.00 ± 0.00 ^Aa^	8.80 ± 0.27 ^Aa^
%2 J	8.80 ± 0.45 ^Aa^	8.80 ± 0.45 ^Aa^	8.70 ± 0.45 ^Aa^	9.00 ± 0.00 ^Aba^	9.00 ± 0.00 ^Aa^	8.90 ± 0.22 ^Aa^
%3 J	8.20 ± 0.27 ^A^	8.00 ± 0.00 ^Aa^	8.00 ± 0.35 ^Aa^	9.00 ± 0.00 ^Aa^	9.00 ± 0.00 ^Aa^	9.00 ± 0.00 ^Aa^
K	7.30 ± 0.45 ^Ab^	7.20 ± 0.84 ^Ab^	6.40 ± 0.55 ^Ac^	8.20 ± 0.45 ^Ab^	8.20 ± 0.45 ^Ab^	7.90 ± 0.22 ^Ab^

C: Carrageenan G: Guar gum X: Xanthan gum J: Gelatin K: Control. A–B statically shows the difference between samples, a–c shows the difference between days.

**Table 14:** Sensorial texture score of cheese samples during storage.

8%Brine				12%Brine		
Samples	1st Day	15th Day	30th Day	1st Day	15th Day	30th Day
%0.5 C	8.66 ± 0.42 ^Aa^	8.50 ± 0.87 ^Aa^	8.30 ± 0.76 ^Aa^	8.54 ± 0.36 ^Ab^	8.70 ± 0.45 ^Aa^	8.50 ± 0.35 ^Aa^
%1.0 C	8.52 ± 0.31 ^Aa^	8.66 ± 0.42 ^Aa^	8.40 ± 0.22 ^Aa^	8.86 ± 0.22 ^Aa^	8.80 ± 0.45 ^Aa^	8.60 ± 0.42 ^Aa^
%1.5 C	8.70 ± 0.45 ^Aa^	8.74 ± 0.42 ^Aa^	8.30 ± 0.27 ^Aa^	8.80 ± 0.27 ^Aa^	8.90 ± 0.22 ^Aa^	8.80 ± 0.27 ^Aa^
%0.25G	6.70 ± 0.76 ^Ab^	6.60 ± 1.14 ^Ac^	6.20 ± 0.76 ^Ab^	8.70 ± 0.27 ^Aa^	8.60 ± 0.42 ^Aa^	8.10 ± 0.22 ^Aa^
%0.50G	7.90 ± 0.42 ^Aa^	7.70 ± 0.45 ^Ab^	7.40 ± 0.42 ^Ab^	8.20 ± 0.27 ^Ab^	8.16 ± 0.32 ^Ab^	7.90 ± 0.42 ^Ab^
%0.75G	8.20 ± 0.27 ^Aa^	8.26 ± 0.49 ^Aa^	8.10 ± 0.42 ^Aa^	8.70 ± 0.45 ^Aa^	8.60 ± 0.55 ^Aa^	8.40 ± 0.65 ^Aa^
%0.25X	8.62 ± 0.28 ^Aa^	8.60 ± 0.55 ^Aa^	8.40 ± 0.42 ^Aa^	8.76 ± 0.25 ^Aa^	8.80 ± 0.27 ^Aa^	8.40 ± 0.22 ^Aa^
%0.50X	8.62 ± 0.36 ^Aa^	8.60 ± 0.55 ^Aa^	8.40 ± 0.42 ^Aa^	8.92 ± 0.11 ^Aa^	9.00 ± 0.00 ^Aa^	8.80 ± 0.27 ^Aa^
%0.75X	8.54 ± 0.46 ^Aa^	8.60 ± 0.55 ^Aa^	8.40 ± 0.42 ^A^	8.80 ± 0.45 ^Aa^	8.80 ± 0.45 ^Aa^	8.70 ± 0.45 ^Aa^
%1 J	8.70 ± 0.27 ^Aa^	8.60 ± 0.55 ^Aa^	8.30 ± 0.45 ^Aa^	9.00 ± 0.00 ^Aa^	9.00 ± 0.00 ^Aa^	8.90 ± 0.22 ^Aa^
%2 J	8.90 ± 0.22 ^Aa^	8.80 ± 0.45 ^Aa^	8.70 ± 0.45 ^Aa^	8.60 ± 0.55 ^Aa^	8.20 ± 1.30 ^Aa^	8.30 ± 0.84 ^Ab^
%3 J	8.70 ± 0.45 ^Aa^	8.60 ± 0.55 ^Aa^	8.50 ± 0.50 ^Aa^	9.00 ± 0.00 ^Aa^	9.00 ± 0.00 ^Aa^	8.90 ± 0.22 ^Aa^
K	7.80 ± 0.27 ^Ab^	7.40 ± 1.34 ^Ab^	6.50 ± 0.50 ^Ab^	8.50 ± 0.50 ^Aa^	8.40 ± 0.65 ^Aa^	8.00 ± 0.35 ^Ab^

C: Carrageenan G: Guar gum X: Xanthan gum J: Gelatin K: Control. A statically shows the difference between samples, a–b shows the difference between days.

**Table 15:** Taste score of cheese samples during storage.

8%Brine				12%Brine		
Samples	1st Day	15th Day	30th Day	1st Day	15th Day	30th Day
%0.5 C	8.76 ± 0.18 ^Aa^	8.74 ± 0.43 ^Aa^	8.54 ± 0.36 ^Aa^	8.56 ± 0.36 ^Aa^	8.54 ± 0.51 ^Aa^	8.40 ± 0.42 ^Aa^
%1.0 C	8.48 ± 0.44 ^Aa^	8.58 ± 0.53 ^Aa^	8.40 ± 0.42 ^Aa^	7.74 ± 0.37 ^Ab^	7.60 ± 0.55 ^Ab^	7.40 ± 0.42 ^Ab^
%1.5 C	8.68 ± 0.24 ^Aa^	8.66 ± 0.42 ^Aa^	8.30 ± 0.27 ^Aa^	8.36 ± 0.5 ^Aa^	8.16 ± 0.71 ^Aa^	8.00 ± 0.61 ^Aa^
%0.25G	7.40 ± 0.82 ^Ab^	7.36 ± 1.16 ^Ab^	6.70 ± 0.57 ^Ac^	8.00 ± 0.35 ^Aab^	7.90 ± 0.74 ^Ab^	7.20 ± 0.27 ^Ab^
%0.50G	8.00 ± 0.35 ^Ab^	7.90 ± 0.22 ^Ab^	7.30 ± 0.27 ^Ab^	7.80 ± 0.27 ^Ab^	7.70 ± 0.45 ^Ab^	7.20 ± 0.27 ^Ab^
%0.75G	8.00 ± 0.61 ^Ab^	7.94 ± 0.82 ^Ab^	7.60 ± 0.65 ^Ab^	7.60 ± 0.42 ^Ab^	7.46 ± 0.55 ^Ab^	7.40 ± 0.65 ^Ab^
%0.25X	8.70 ± 0.35 ^Aa^	8.80 ± 0.45 ^Aa^	8.70 ± 0.45 ^Aa^	7.70 ± 0.57 ^Ab^	7.70 ± 0.84 ^Ab^	7.50 ± 0.50 ^Aa^
%0.50X	8.60 ± 0.65 ^Aa^	8.60 ± 0.89 ^Aa^	8.30 ± 0.84 ^Aa^	8.10 ± 0.42 ^Aa^	8.10 ± 0.74 ^Aa^	7.90 ± 0.55 ^Aa^
%0.75X	8.30 ± 0.45 ^Aa^	8.30 ± 0.45 ^Aa^	8.10 ± 0.22 ^Aa^	7.30 ± 0.57 ^Ab^	7.00 ± 0.71 ^Ac^	6.70 ± 0.45 ^Ac^
%1 J	8.90 ± 0.22 ^Aa^	8.80 ± 0.45 ^Aa^	8.40 ± 0.42 ^Aa^	7.90 ± 0.65 ^Aa^	7.60 ± 0.89 ^Ab^	7.50 ± 0.71 ^Ab^
%2 J	8.70 ± 0.45 ^Aa^	8.60 ± 0.55 ^Aa^	8.40 ± 0.55 ^Aa^	8.10 ± 0.65 ^Aa^	7.90 ± 0.89 ^Aa^	7.70 ± 0.67 ^Aa^
%3 J	8.20 ± 0.76 ^Aa^	8.00 ± 1.00 ^Ab^	7.80 ± 0.76 ^Ab^	8.50 ± 0.35 ^Aa^	8.40 ± 0.42 ^Aa^	7.90 ± 0.22 ^Aa^
K	8.60 ± 0.55 ^Aa^	8.60 ± 0.55 ^Aa^	7.40 ± 0.65 ^Ab^	7.30 ± 0.67 ^Ab^	7.00 ± 1.22 ^Ab^	6.60 ± 0.65 ^Ac^

C: Carrageenan G: Guar gum X: Xanthan gum J: Gelatin K: Control. A statically shows the difference between samples, a–b shows the difference between days.

**Table 16:** General acceptance score of cheese samples during storage.

8%Brine				12%Brine		
Samples	1st Day	15th Day	30th Day	1st Day	15th Day	30th Day
%0.5 C	8.66 ± 0.15 ^Aa^	8.60 ± 0.38 ^Aa^	8.46 ± 0.29 ^Aa^	8.60 ± 0.38 ^Aa^	8.62 ± 0.39 ^Aa^	8.36 ± 0.35 ^Aa^
%1.0 C	8.50 ± 0.37 ^Aa^	8.52 ± 0.48 ^Aa^	8.30 ± 0.27 ^Aa^	8.12 ± 0.44 ^Aa^	8.20 ± 0.57 ^Aa^	8.00 ± 0.35 ^Aa^
%1.5 C	8.52 ± 0.33 ^Aa^	8.50 ± 0.46 ^Aa^	8.30 ± 0.27 ^Aa^	8.56 ± 0.38 ^Aa^	8.58 ± 0.40 ^Aa^	8.40 ± 0.22 ^Aa^
%0.25G	6.50 ± 0.71 ^Ac^	6.80 ± 1.3 ^Ac^	6.20 ± 0.76 ^Ac^	8.50 ± 0.50 ^Aa^	8.46 ± 0.55 ^Aa^	7.70 ± 0.27 ^Ab^
%0.50G	7.90 ± 0.22 ^Ab^	7.76 ± 0.43 ^Ab^	7.30 ± 0.45 ^Ab^	8.10 ± 0.22 ^Aa^	7.98 ± 0.04 ^Ab^	7.60 ± 0.22 ^Ab^
%0.75G	8.20 ± 0.57 ^Aa^	8.06 ± 0.61 ^Aa^	7.90 ± 0.22 ^Aab^	8.35 ± 0.22 ^Aa^	8.25 ± 0.25 ^Aa^	8.00 ± 0.35 ^Aa^
%0.25X	8.70 ± 0.45 ^Aa^	8.76 ± 0.43 ^Aa^	8.60 ± 0.42 ^Aa^	8.26 ± 0.37 ^Aa^	8.40 ± 0.55 ^Aa^	8.10 ± 0.22 ^Aa^
%0.50X	8.66 ± 0.42 ^Aa^	8.76 ± 0.43 ^Aa^	8.56 ± 0.38 ^Aa^	8.48 ± 0.48 ^Aa^	8.58 ± 0.53 ^Aa^	8.28 ± 0.41 ^Aa^
%0.75X	8.50 ± 0.35 ^Aa^	8.50 ± 0.35 ^Aa^	8.30 ± 0.27 ^Aa^	7.90 ± 0.22 ^Ab^	7.86 ± 0.74 ^Ab^	7.60 ± 0.42 ^Ab^
%1 J	8.80 ± 0.27 ^Aa^	8.70 ± 0.45 ^Aa^	8.30 ± 0.45 ^Aa^	8.40 ± 0.42 ^Aa^	8.30 ± 0.45 ^Aa^	8.10 ± 0.22 ^Aa^
%2 J	8.70 ± 0.45 ^Aa^	8.60 ± 0.55 ^Aa^	8.30 ± 0.45 ^Aa^	8.20 ± 0.57 ^Aa^	8.10 ± 0.74 ^Aa^	7.90 ± 0.55 ^Ab^
%3 J	8.30 ± 0.67 ^Aa^	8.00 ± 1.00 ^Ab^	7.70 ± 0.45 ^Ab^	8.90 ± 0.22 ^Aa^	8.90 ± 0.22 ^Aa^	8.20 ± 0.27 ^Aa^
K	7.60 ± 0.55 ^Ab^	7.40 ± 0.82 ^Ab^	7.00 ± 0.35 ^Ab^	7.90 ± 0.74 ^Ab^	7.70 ± 1.10 ^Ab^	7.00 ± 0.61 ^Ab^

C: Carrageenan G: Guar gum X: Xanthan gum J: Gelatin K: Control. A statically shows the difference between samples, a–c shows the difference between days.

## References

[1] Gahun Y. (1978). Peynire tuz geçişini etkileyen faktörler. J. Food.

[2] Guinee T.P. (2004). Salting and the role of salt in cheese. Int. J. Dairy Technol..

[3] Özer B., Atasoy F., Akın S. (2002). Some properties of Urfa Cheese (A Traditional White-Brined Turkish Cheese) produced from bovine and ovine milks. Int. J. Dairy Technol..

[4] Hayaloğlu A.A., Güven M., Fox P.F. (2002). Microbiological, biochemical and technological properties of Turkish White Cheese ‘Beyaz Peynir’. Int. Dairy J..

[5] Güler Z., Uraz T. (2004). Relationships between proteolytic and lipolytic activity and sensory properties (taste and odour) of traditional Turkish white cheese. Dairy Technol..

[6] Prasad N., Alvarez V.B. (1999). Effect of salt and chymosin on the physicochemical properties of feta cheese during ripening. J. Dairy Sci..

[7] Cankurt H. (2015). Bazı bitki su ve uçucu yağların blok tipi eritme peyniri ve beyaz peynirin çeşitli özellikleri üzerine etkisi. Ph.D. Thesis.

[8] Uraz G.T., Yıldız F. (2004). Salamura sıcaklığının beyazpeynirde tuz geçişi üzerine etkisi.

[9] Fox P.F. (1987). Cheese: Chemistry, Physics and Microbiology.

[10] Koçak C., Gürsel A., Ergül E., Gürsoy A. (1990). Farklı asitlikteki salamurada bekletmenin peynire tuz geçişi üzerine etkisi. J. Food.

[11] Turantaş F., Ünlütürk A., Göktan D. (1989). Microbiological and compositional status of turkish white cheese. Int. J. Food Microbiol..

[12] Üçüncü M. (1983). Beyaz peynir yapımında tuz ve tuzlama sorunları.

[13] (2010). Türkiye aşırı tuz tüketiminin azaltılması programı 2011–2015.

[14] Ahmed N.H., El Soda M., Hassan A.N., Frank J. (2005). Improving the textural properties of an acid-coagulated (karish) cheese using exoploysaccharide producing cultures. Lwt-Food Sci. Technol..

[15] De Man J.C., Rogosa M., Sharpe M.E. (1960). A medium for the cultivation of Lactobacilli. J. Appl. Bacteriol..

[16] Terzaghi B.E., Sandine W.E. (1975). Improved medium for lactic streptococci and their bacteriophages. Appl. Microbiol..

[17] Halkman K. (2005). Merck gıda mikrobiyolojisi uygulamaları. Ankara. http://www.mikrobiyoloji.org.

[18] Yetim H. (2001). Gıda Analizleri.

[19] (2000). SAS/STAT User’s Guide (6, 03).

[20] Ayad E.H.E., Verheul A., Wouters J.T.M., Smit G. (2001). Population dynamics of lactoccoci from industrial, artisanal and non-dairy origins in defined strain starters for gouda type cheese. Int. Dairy J..

[21] Ayad E.H.E., Verheul A., Wouters J.T.M., Smit G. (2002). Antimicrobial-producing wild *lactococcus* isolated from artisanal and non- dairy origins. Int. Dairy J..

[22] Şengül M., Erkaya T., Fırat N. (2010). Çiğ ve pastörize sütten üretilen kaşar peynirlerinin olgunlaşma süresince bazı mikrobiyolojik özelliklerinin karşılaştırılması. Atatürk Univ. J. Agric. Fac..

[23] Kesenkaş H., Dinkçi N., Kınık Ö. (2012). Farklı işletmelerde üretilen köy peynirlerinin özellikleri. Ege Univ., J. Agric. Fac..

[24] Şener L.G. (2012). Beyaz peynir üretiminde transglutanminaz enzimi kullanım olanakları. Masters’s Thesis.

[25] Öztürk İ. (2013). Geleneksel fermente sucuklardan izole edilen mayaların tanımlanması, teknolojik ve fonksiyonel özelliklerinin belirlenmesi ve sucuk üretimine uygun izolatların seçilmesi. Ph.D. Thesis.

[26] Koçak C. (1988). Süte uygulanan ısıl işlemlerin sütün peynir mayası ile pıhtılaşma yeteneğine etkisi. 1.

[27] Akalın A.Ş., Karaman A.D. (2011). Influence of packaging systems on the biochemical characteristics and volatile compounds of industrially produced Turkish White cheese. J. Food Biochem..

[28] Kaya S. (2002). Effect of salt on hardness and whiteness of Gaziantep cheese during short-term brining. J. Food Eng..

[29] Kırkın C., Gunes G., Kılıc-Akyılmaz M. (2013). Preservation of precut white cheese by modified atmosphere packaging. Int. J. Dairy Technol..

[30] Şahingil D., Hayaloğlu A.A., Simsek O., Ozer B. (2014). Changes in volatile composition, proteolysis and textural and sensory properties of whie-brined cheese: Effects of ripening temperature and adjuct culture. Dairy Sci. Technol..

[31] Koyuncu M. (2017). Beyaz peynirde yumuşama kusurunun farklı üretim ve olgunlaştırma uygulamalarıyla önlenmesi üzerine araştırmalar. Ph.D. Thesis.

[32] Solorza J.F. (1996). Studies of the Effect of Different Factors on the Rheological Properties of a Soft Cheese. Ph.D. Thesis.

[33] Ghoddushi H.B. (1996). Some Aspects of the Enumeration of Bifidobacteria in White Brined Cheese. Ph.D. Thesis.

[34] Yerlikaya O. (2008). Kaparili beyaz peynir üretimi ve kalite özellikleri üzerine bir araştırma. Masters’s Thesis.

[35] Zoon P. (1991). The relation between ınstrumental and sensory evaluation of the rheological and fracture properties of cheese. Rheological and Fracture Properties of Cheese.

[36] Van Vliet T. (1991). Terminology to be used in cheese rheology. Rheological and Fracture Properties of Cheese.

[37] Imoto E.M., Lee C.H., Rha C. (1979). Effect of compression ratio on the mechanical properties of cheese. J. Food Sci..

[38] Qvist K.B. (1987). Objective and Sensory Assessment of Texture of Danbo Cheese Made from Milk Concentrated 2-Fold Using Ultrafiltration.

